# Lipopolysaccharide Disrupts the Milk-Blood Barrier by Modulating Claudins in Mammary Alveolar Tight Junctions

**DOI:** 10.1371/journal.pone.0062187

**Published:** 2013-04-23

**Authors:** Ken Kobayashi, Shoko Oyama, Atsushi Numata, Md. Morshedur Rahman, Haruto Kumura

**Affiliations:** Laboratory of Dairy Food Science, Research Faculty of Agriculture, Hokkaido University, Sapporo, Japan; Auburn University, United States of America

## Abstract

Mastitis, inflammation of the mammary gland, is the most costly common disease in the dairy industry, and is caused by mammary pathogenic bacteria, including *Escherichia coli*. The bacteria invade the mammary alveolar lumen and disrupt the blood-milk barrier. In normal mammary gland, alveolar epithelial tight junctions (TJs) contribute the blood-milk barrier of alveolar epithelium by blocking the leakage of milk components from the luminal side into the blood serum. In this study, we focused on claudin subtypes that participate in the alveolar epithelial TJs, because the composition of claudins is an important factor that affects TJ permeability. In normal mouse lactating mammary glands, alveolar TJs consist of claudin-3 without claudin-1, -4, and -7. In lipopolysaccharide (LPS)-induced mastitis, alveolar TJs showed 2-staged compositional changes in claudins. First, a qualitative change in claudin-3, presumably caused by phosphorylation and participation of claudin-7 in alveolar TJs, was recognized in parallel with the leakage of fluorescein isothiocyanate-conjugated albumin (FITC-albumin) via the alveolar epithelium. Second, claudin-4 participated in alveolar TJs with claudin-3 and claudin-7 12 h after LPS injection. The partial localization of claudin-1 was also observed by immunostaining. Coinciding with the second change of alveolar TJs, the severe disruption of the blood-milk barrier was recognized by ectopic localization of β-casein and much leakage of FITC-albumin. Furthermore, the localization of toll-like receptor 4 (TLR4) on the luminal side and NFκB activation by LPS was observed in the alveolar epithelial cells. We suggest that the weakening and disruption of the blood-milk barrier are caused by compositional changes of claudins in alveolar epithelial TJs through LPS/TLR4 signaling.

## Introduction

The mammary gland is a highly specialized organ in female mammals and provides nutrition to the suckling infant. Milk secretion is maintained by the suckling infant from parturition until weaning in the normal mammary gland. However, mastitis, the inflammation of mammary glands resulting from pathogenic bacterial infection, disrupts normal milk secretion from alveolar epithelial cells [1]. Individuals with infected mammary glands present mastitis-specific symptoms such as breast tenderness, fever, and qualitative and quantitative changes of milk [2], [3]. Bacterial infection also causes the disruption of directionally controlled milk secretion. For example, α-lactalbumin and casein leak into blood serum in the case of intramammary infusion of bacteria or sterile endotoxin [4], [5]. Burton and Erskine have suggested that the leakage of blood constituents during the early acute stage of mammary inflammation occurs because of an alteration in the blood-milk barrier [6]. However, how infected bacteria cause changes in the blood-milk barrier in mastitis remains unclear.

Several studies have indicated that the mammary alveolar tight junctions (TJs) are impermeable during lactation and thus allow milk to be secreted from the apical membrane without the leakage of milk components from the lumen via paracellular pathways in goats and cows [7], [8], [9]. Alveolar TJs contribute to the blood-milk barrier by blocking the leakage of milk components from the luminal side into the blood serum. TJs are specific structures localized at the most apical regions of epithelial cell membranes that restrict the paracellular flow of aqueous molecules, ions, water, and microbes [10], [11]. Interestingly, although TJs function as an impermeable barrier between neighboring epithelial cells against microbes, some microbes or their endotoxins are able to disrupt TJs to infect the host and spread the infection [12], [13], [14].

TJ structure is constructed from multiple components such as occludin, zonula occludens-1, junctional adhesion molecules, and claudins in mammals [15]. In particular, at least 24 subtypes have been reported to comprise the claudin family, and specific combinations of them are observed in several epithelial tissues in mice and humans [16]. The composition of claudins is a determinant of TJ permeability [17]. For example, transfection of claudin-2 into MDCK I cells, which endogenously express claudin-1 and -4, induces leaky TJs, compared to TJs of normal MDCK I cells [18]. In mouse mammary glands, structural changes of TJs occur around parturition, during lactation, and after weaning [19]. The full functional integrity of TJs is established shortly after parturition, and they remain closed throughout lactation [20], [21]. Our previous study indicated that the alveolar TJs show drastically increased claudin-4 levels and qualitative changes of claudin-3 that are presumably caused by phosphorylation before parturition and after weaning in mice [22]. Inflammation also induces changes in claudin compositions with weakened TJs in goats and cows [23], [24]. These reports suggest the possibility that the blood-milk barrier in mastitis is disrupted by compositional changes of claudins in the alveolar epithelial TJs.

Mastitis is caused by several mammary pathogenic bacteria including *Escherichia coli* [25]. Intramammary administration of lipopolysaccharide (LPS) as a typical mastitis endotoxin from *E. coli* is a well-established method for experimental induction of mastitis under defined conditions to study the immune response of the mammary gland in cows [26], [27], [28]. The claudin subtypes that exist in mammary glands, including claudin-1, -3, -4, and -7, have been reported to show distinct expression and localization patterns that are modified by stimuli of sucking, weaning, and parturition in mice [22], [29], [30]. In this study, we injected LPS into the mouse mammary glands to induce artificial mastitis, and we investigated the influences of LPS on behaviors of claudin-1, -3, -4, and -7 with respect to TJ permeability.

## Materials and Methods

### Animals

Pregnant ICR mice were purchased from Japan SLC, Inc. (Shizuoka, Japan). After parturition, the lactating mouse was kept with suckling neonatal pups. LPS that originated from *E. coli* 0111:B4 (L3024, Sigma, St. Louis, MO) and was solubilized in 0.5 mM CaCl_2_ and 0.5 mM MgCl_2_–containing phosphate-buffered saline (mPBS) at a concentration of 0.2 mg/mL. LPS (20 µg) was injected into the fourth inguinal mammary gland via teat canal on day 10 of lactation under anesthesia with pentobarbital. Three, six, or twelve hours after LPS injection, the mice were decapitated, and the mammary glands were dissected. In each of the experiments, the dissected mammary glands were washed with mPBS and then used immediately. In this study, we used the mammary glands without injection treatment as a control (0 h of LPS injection). All experimental procedures in this study were approved by Animal Resource Committee of Hokkaido University, and were conducted in accordance with Hokkaido University guidelines for the care and use of laboratory animals.

### Materials

LPS and fluorescein isothiocyanate-conjugated albumin (FITC-albumin) were purchased from Sigma-Aldrich. The following antibodies were used as primary antibodies for immunological studies: rabbit polyclonal antibodies against claudin-1, -3, -4, and -7 (Invitrogen/Zymed Laboratories, San Francisco, CA); NFκB (Cell Signaling Technology, Danvers, MA); toll-like receptor 4 (TLR4; Santa Cruz Biotechnology, Santa Cruz, CA); and mouse monoclonal antibodies against occludin (Invitrogen/Zymed Laboratories) and pan-keratin (Sigma-Aldrich). Secondary Alexa Fluor 488-conjugated goat anti-rabbit, Alexa Fluor 546-conjugated goat anti-mouse, and Alexa Fluor 546-conjugated rabbit anti-goat antibodies were purchased from Invitrogen/Molecular Probes (Eugene, OR).

### FITC-albumin Treatment to Evaluate Alveolar TJ Permeability

To visualize alveolar TJ permeability, the mammary glands without injection and 3, 6, and 12 h after LPS injection were treated with FITC-conjugated albumin according to Nguyen’s method [31]. In brief, a mouse was deeply anesthetized with pentobarbital and the fourth mammary gland was surgically exposed. The mammary gland was immersed in mPBS containing 3 mg/mL FITC-albumin to expose the interstitial side of the alveolar epithelial cells. After treatment with FITC-albumin for 10 min, the mammary gland was washed in mPBS 3 times and then immersed in mPBS containing 4% paraformaldehyde for 10 min. The pre-fixed mammary gland was embedded in optimal cutting temperature (OCT) compounds and was frozen with liquid nitrogen, after which 5-µm cryosections were obtained. The cryosections were post-fixed with PBS containing 1% paraformaldehyde, stained with 4′,6-diamidino-2-phenylindole (DAPI), and mounted with fluoromount (Diagnostic BioSystems, Pleasanton, CA).

### Isolation of Mammary Alveolar Epithelial Cells

Mammary glands were harvested from lactating mice and minced with a scalpel. Minced mammary glands were treated with 2 mg/mL collagenase type I (Worthington Biochemical Corporation, Lakewood, NJ) containing RPMI-1640 medium for 2 h at 37°C. After gentle pipetting with a Pasteur pipette, mammary alveoli were separated from fat and single cells by centrifugation at 100×*g* for 10 min in 5% BSA containing RPMI-1640. Mammary alveoli were treated with RPMI-1640 containing 0.1% trypsin for 5 min at room temperature to remove the myoepithelial cells and were then centrifuged at 100×*g* for 10 min in 5% BSA. The isolated mammary alveoli were cultured for 5 days in growth medium containing 5 µg/mL insulin, 10 ng/mL epidermal growth factor, and 10% fetal bovine serum (FBS). After 5 days of culture, the growth medium was removed, and the cells were cultured in differentiation medium containing 5 µg/mL insulin, 1 µM dexamethasone, 10 µg/mL prolactin, and 1% FBS for 3 days. After cultivation, the alveolar epithelial cells were fixed with 1% paraformaldehyde in mPBS and were used for Immunofluorescence staining.

### Immunofluorescence Staining

The mammary glands were fixed with 4% paraformaldehyde in PBS, pH 7.4, for 1 day at 4°C and embedded in paraffin. The embedded samples were sliced into 5-µm sections, and the sections were deparaffinized and hydrated. The sections were incubated with PBS containing 5% bovine serum albumin (BSA) to block nonspecific interactions and were then treated with primary antibodies diluted in the blocking solution overnight at 4°C. After the sections were washed with PBS, they were exposed to secondary antibodies for 1 h at room temperature in the blocking solution. Controls were treated in the same manner, except for the exclusion of the primary antibodies. Images of the stained sections were obtained using a confocal laser-scanning microscope (TCS SP5; Leica, Mannheim, Germany). The position of the apical junction was determined by the localization of occludin, a representative TJ component that is mainly localized to the apical junction [32].

### Western Blotting Analysis

The samples were electrophoresed using a 12% SDS-polyacrylamide gel and transferred onto polyvinylidene difluoride membranes (Bio-Rad Laboratories, Hercules, CA). The membranes were blocked for 1 h with PBS containing 3% nonfat dried milk and 0.05% Tween 20 and then incubated overnight at 4°C with primary antibodies diluted in PBS containing 5% BSA. Subsequently, the membranes were washed in PBS containing 0.05% Tween 20 and incubated for 45 min at room temperature with the appropriate secondary horseradish peroxidase-conjugated antibodies diluted in PBS containing 3% nonfat dried milk and 0.05% Tween 20. The immunoreactive bands were detected using Luminate Forte Western HRP substrate (Millipore, Billerica, MA). The volume of the protein bands was measured by a Bio-Rad ChemiDoc™ EQ densitometer and a Bio-Rad Quantity One® software (Bio-Rad).

### Preparation of Triton X-100–soluble and Triton X-100–insoluble Protein Fractions

The minced mammary gland was lysed in Triton X-100 buffer containing 1% Triton X-100, 100 mM NaCl, 10 mM N-(2-hydroxyethyl)-piperazine-N′-2-ethanesulfonic acid (pH 7.4), 2 mM EDTA, a phosphatase inhibitor mixture (PhosStop, Roche, Mannheim, Germany), and a protease inhibitor mixture (complete mini, Roche) and then passed 30 times through a 21-gauge needle. The lysates were then centrifuged at 15,000× *g* for 30 min at 4°C. The supernatant was considered to be the Triton X-100–soluble fraction. The pellet was solubilized in Triton X-100 buffer containing 1% SDS by using an ultrasonic disintegrator and then centrifuged at 15,000× *g* for 5 min at 4°C. The supernatant was designated as the Triton X-100–insoluble fraction. As a sample for western blotting, each fraction was diluted with an equal volume of sample buffer (100 mM Tris (pH 6.8), 100 mM dithiothreitol, 2% SDS, 0.2% bromophenol blue, and 20% glycerol), incubated for 10 min at 70°C, and stored at −20°C.

### Statistical Analysis

Data are expressed as mean (SD). The statistical significance of differences between mean values was tested using a Student's t test. Differences were considered significant at p-value <0.05. All experiments were performed a minimum of 4 times using different mice to ensure reproducibility.

## Results

### LPS Disrupts the Blood-milk Barrier

**Figure 1:**
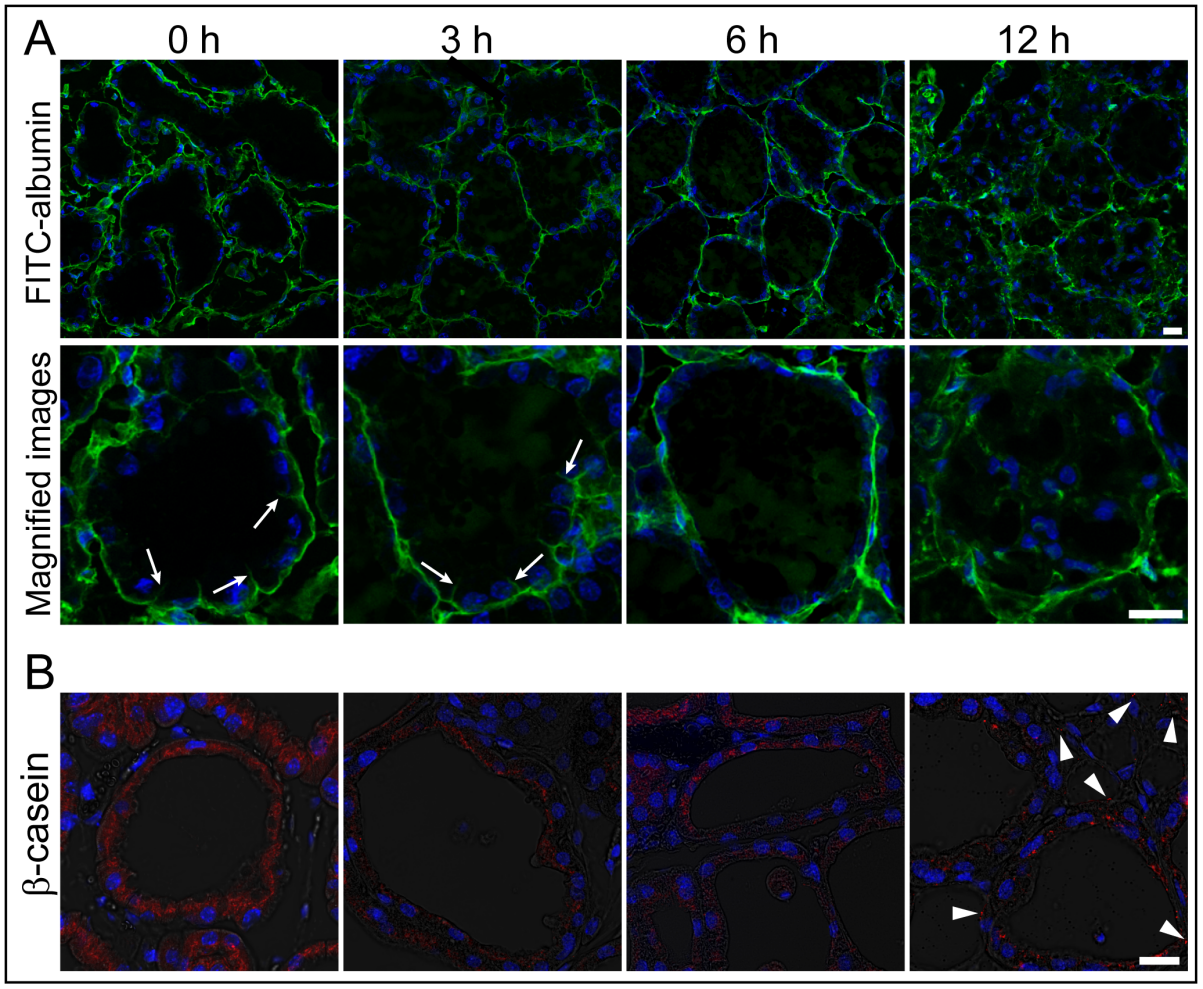
LPS increases the permeability of the alveolar epithelium. Mammary glands non-treated (0 h) and 3, 6, and 12 h after lipopolysaccharide (LPS) injection were immersed in FITC-albumin containing 0.5 mM CaCl_2_ and 0.5 mM MgCl_2_–containing phosphate-buffered saline (mPBS), and the localization of FITC-albumin was observed after cutting the frozen sections (A). Green and blue show FITC-albumin and nuclei (4′,6-diamidino-2-phenylindole, DAPI), respectively. FITC-albumin was observed in the alveolar lumen of LPS-injected mammary glands. Arrows show the localization of FITC-albumin in the interfacing regions of alveolar epithelial cells. (B) The paraffin sections were stained with antibodies to β-casein (red). Arrowheads show localization of β-casein on the interstitial side. Scale bars: 20 µm.

To evaluate the influences of LPS on the blood-milk barrier, the mammary glands non-treated and 3, 6, and 12 h after LPS injection were immersed in FITC-albumin containing saline, and the leakage of FITC-albumin from the interstitial side into the alveolar lumen was visualized. The mammary glands did not show any fluorescent reactions in the alveolar lumen before LPS injection although FITC-albumin was obviously localized on the interstitial side containing the intercellular regions of the neighboring alveolar epithelial cells (Fig. 1A). FITC-albumin was thinly distributed in parts of the mammary alveolar lumen 3 h after LPS injection, in addition to the interstitial side containing the intercellular regions of alveolar epithelial cells. The FITC-positive reactions in the alveolar lumen drastically increased 12 h after LPS injection, whereas FITC-albumin was not observed in the intercellular regions of alveolar epithelial cells 6 and 12 h after LPS injection.

Beta-casein is a major milk component, and it forms casein micelles of diameter approximately 100 nm along with other types of casein and fat in cows [33]. In the normal lactating mammary gland, β-casein was observed on the luminal side containing alveolar epithelial cells but not on the interstitial side (Fig. 1B). The presence of β-casein on the interstitial side was not confirmed 3 or 6 h after LPS injection. Twelve hours after LPS injection, β-casein was detected on the interstitial side of the mammary alveolar epithelium.

### LPS Influences the Expression levels of Claudin-1, -3, -4, and -7

**Figure 2:**
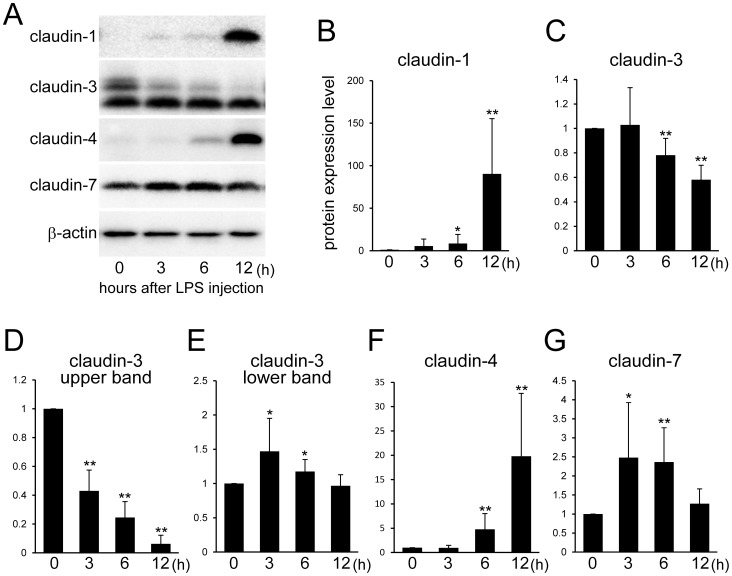
Expression changes of claudin-1, -3, -4, and -7 after LPS injection. (A) Results of a western blot analysis of claudin-1, -3, -4, and -7 and β-actin in the mammary glands non-treated (0 h) and 3, 6, and 12 h after LPS injection. (B) The relative expression levels of claudin-1, -3 (total, upper band, lower band), -4, and -7 were analyzed by densitometry. The upper band of claudin-3 gradually disappeared after LPS injection. Beta-actin was used as a normalization control. Data represent mean (SD) (n  = 6). *, p<0.05; **, p<0.005 vs. 0 h.

To investigate the influences of LPS on expression levels of claudin-1, -3, -4, and -7, western blotting was performed. Claudin-1 was barely detected in the mammary gland before LPS injection (Fig. 2A). The expression of claudin-1 was induced by LPS injections, and densitometry analysis showed approximately 80-fold increases of claudin-1 12 hours after injection (Fig. 2B). In the normal mammary gland, claudin-3 was detected as at least 2 bands (upper and lower bands), which was similar to our previous finding [22]. The size of the upper band of claudin-3 rapidly decreased 3 h after LPS injection although the size of the lower band showed a gradual decrease. The total volume of claudin-3 significantly decreased 6 h after LPS injection, and the size of the upper and the lower band of claudin-3 showed a significant decrease and increase 3 h after LPS injection, respectively (Fig. 2C–E). Claudin-4 significantly increased approximately 5- and 20-fold 6 and 12 h after LPS injection, respectively (Fig. 2F). Claudin-7 level significantly increased 3 and 6 h after LPS injection; however, the mammary gland 12 h after LPS injection did not show a significant difference from that without LPS injection (Fig. 2G).

### LPS Changes the Localization of Claudin-1, -3, -4, and -7

To investigate the temporal and spatial changes of claudin-1, -3, -4, and -7 in the mammary alveolar epithelium non-treated and 3, 6, and 12 h after LPS injection, immunostaining was performed. Occludin was double-stained with claudins to indicate the apical-most regions, where TJs are localized [32].

**Figure 3:**
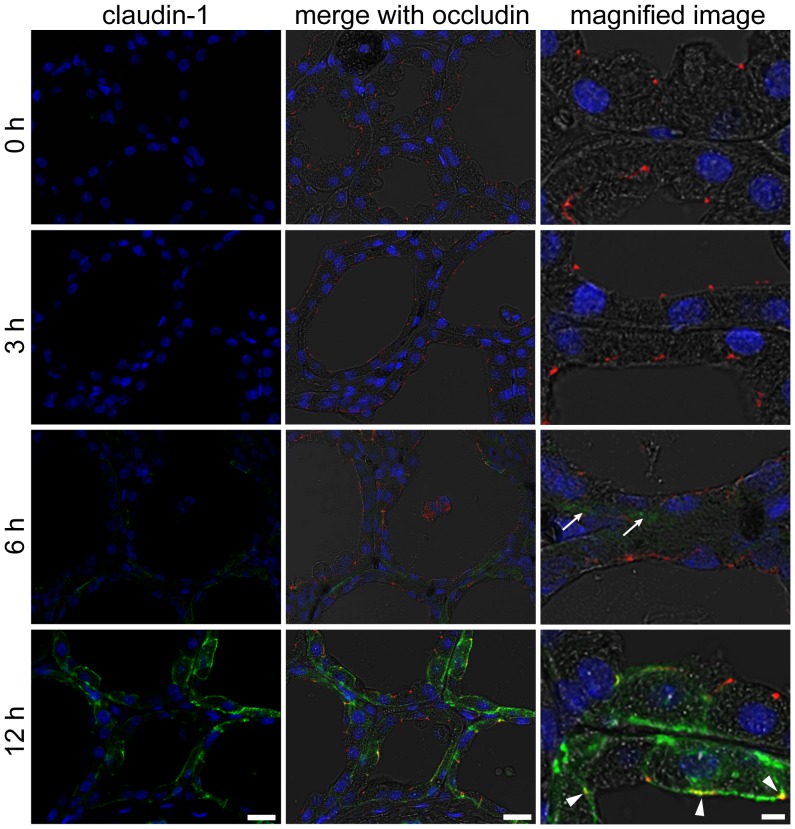
LPS induces claudin-1 in the mammary alveolar epithelium. The left column shows the immunostaining images of claudin-1 (green) and nuclear staining with DAPI (blue) in mammary glands non-treated (0 h) and 3, 6, and 12 h after LPS injection. The middle and right columns show the merged images with occludin (red) and bright field. Arrows indicate claudin-1–positive regions in basolateral membranes 6 h after LPS injection. Arrowheads indicate the localization of claudin-1 in the apical membrane 12 h after LPS injection. Scale bars: 20 µm (left and middle columns) and 5 µm (right column).

A positive reaction to claudin-1 was not observed in the mammary alveolar epithelium before LPS injection or 3 h after LPS injections (Fig. 3). Six hours after LPS injection, some of alveolar epithelial cells showed a weak positive reaction to claudin-1 in the basolateral membrane. Claudin-1–positive cells drastically increased 12 h after injection, and about half the number of the alveolar epithelial cells showed localization of claudin-1 in the basolateral membranes. Parts of the alveolar epithelial cells also showed colocalization of claudin-1 and occludin at the apical-most regions.

**Figure 4:**
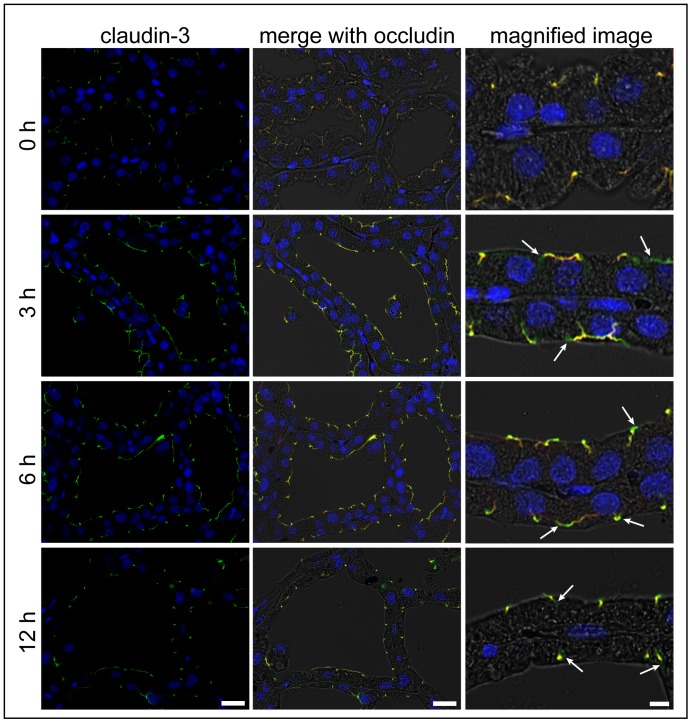
Claudin-3 localization around the apical-most regions before and after LPS injection. The left column shows the immunostaining images of claudin-3 (green) and nuclear staining with DAPI (blue) in mammary glands non-treated (0 h) and 3, 6, and 12 h after LPS injection. The middle and right columns show the merged images with occludin (red) and bright field. Arrows indicate the localization of claudin-3 in the absence of occludin around the apical membrane after LPS injection. Scale bars: 20 µm (left and middle columns) and 5 µm (right column).

Claudin-3 colocalized with occludin at the apical-most regions in the mammary alveolar epithelium before LPS injection without any differences between their localization patterns (Fig. 4). Three hours after LPS injection, claudin-3 was mainly colocalized with occludin at the apical-most regions, and the single localization of claudin-3 without occludin was also observed around the apical-most regions. Such different localizations between claudin-3 and occludin were also observed in the alveolar epithelial cells 6 and 12 h after LPS injection.

**Figure 5:**
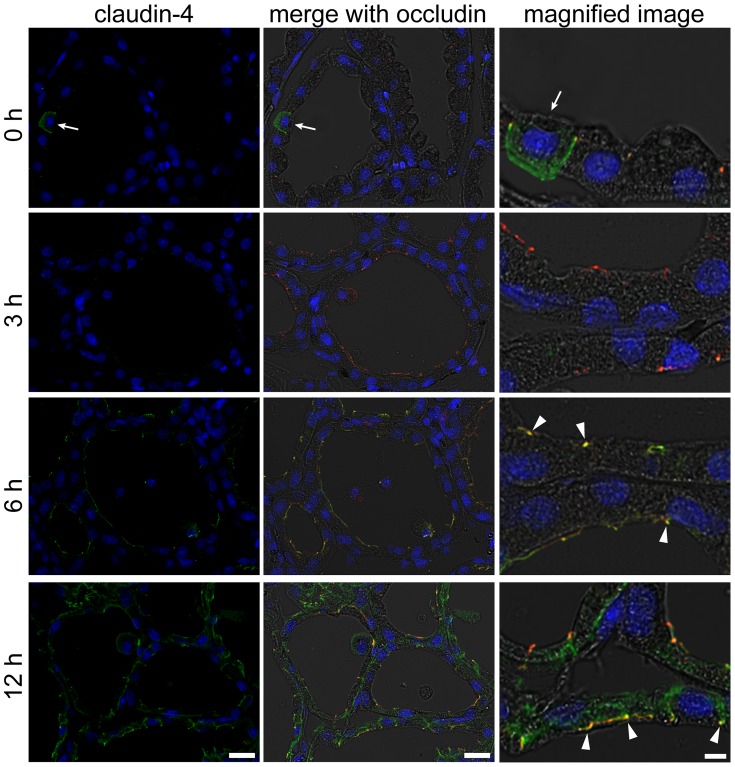
LPS induces claudin-4 expression in the mammary alveolar epithelium. The left column shows the immunostaining images of claudin-4 (green) and nuclear staining with DAPI (blue) in mammary glands non-treated (0 h) and 3, 6, and 12 h after LPS injection. The middle and right columns show the merged images with occludin (red) and bright field, respectively. Scale bars: 20 µm (left and middle columns) and 5 µm (right column). Arrows indicate claudin-4–positive cells, which are rarely observed in the mammary alveolar epithelium. Arrowheads indicate the colocalization of claudin-4 and occludin at the apical-most regions.

Claudin-4 was not detected in most of the alveolar epithelial cells in the normal lactating mammary glands, whereas very few cells (approximately 1 cell per 20 mammary alveoli) showed a positive reaction to claudin-4 (Fig. 5). A similar localization pattern was observed in the mammary gland 3 h after LPS injection. Six hours after LPS injection, claudin-4 was observed at the apical-most regions indicated by occludin and around the apical-most regions in parts of the alveolar epithelial cells. Twelve hours after LPS injection, a clear localization of claudin-4 was observed in the basolateral membrane of all of the alveolar epithelial cells, and parts of the cells showed localization of claudin-4 at the apical-most regions.

**Figure 6:**
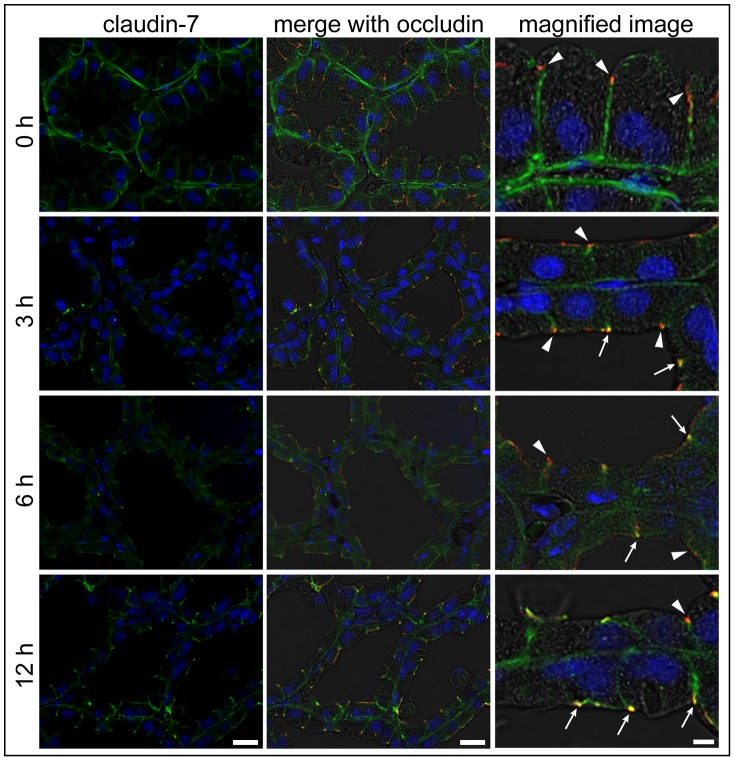
LPS induces the translocation of claudin-7 from the basolateral membrane to the apical-most regions. The left column shows the immunostaining images of claudin-7 (green) and nuclear staining with DAPI (blue) in mammary glands non-treated (0 h) and 3, 6, and 12 h after LPS injection. The middle and right columns show the merged images with occludin (red) and bright field, respectively. Scale bars: 20 µm (left and middle columns) and 5 µm (right column). Arrows indicate claudin-7–positive regions at the apical-most regions in the mammary alveolar epithelial cells with LPS injection. Arrowheads indicate the apical-most regions without claudin-7.

In normal lactating mammary glands, claudin-7 was localized in the basolateral membrane (Fig. 6). In particular, the localization of claudin-7 along the basal membrane of the alveolar epithelial cells was clearly observed as a line. Three hours after LPS injection, parts of alveolar epithelial cells showed localization of claudin-7 at the apical-most regions; on the other hand, the staining intensity of claudin-7 in the basolateral membrane weakened. Similar localization patterns were observed in the mammary gland 6 and 12 h after LPS injection. On the other hand, vehicle (mPBS)-injected mammary glands did not induce any changes of claudin localization patterns compared to mammary glands without injection treatment (Figure S1).

### LPS Changes the Cellular Distribution of Claudins Detected by Detergent Solubility

The intracellular distribution of claudins in the mammary glands non-treated and 3, 6, and 12 h after LPS injection was examined by detergent insolubility. Detergent-soluble fractions show the representative cytosolic distribution or loose association with the basolateral membrane, and detergent-insoluble fractions show the representative TJ strand distribution at the apical-most regions [34]. To confirm the claudins participating in TJs, western-blotting experiments of the detergent-insoluble fraction were performed.

**Figure 7:**
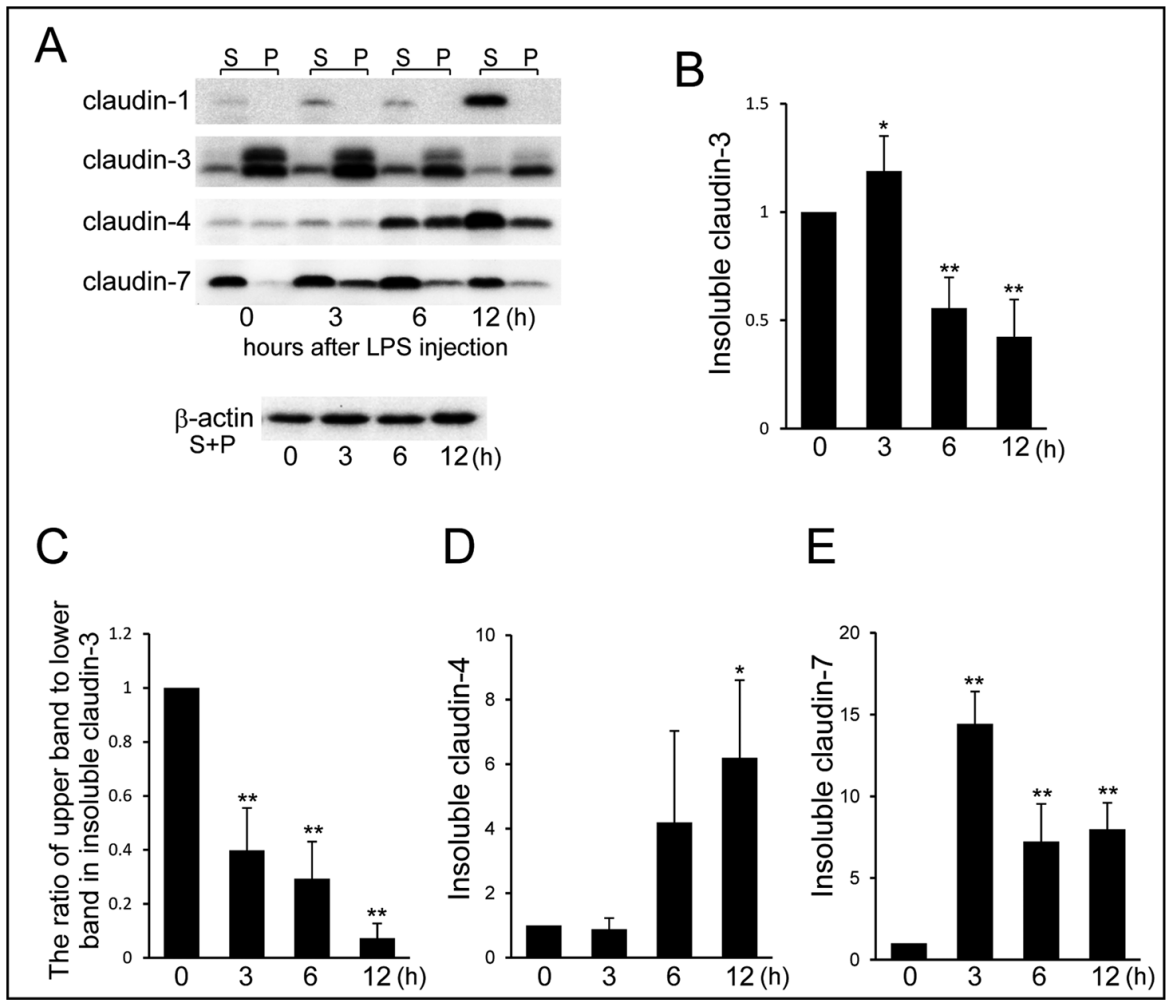
Influences of LPS on the detergent solubility of claudin-1, -3, -4, and -7. (A) LPS was injected into the mammary glands on day 10 of lactation; the detergent-soluble (S) and detergent-insoluble (P) fractions of the mammary glands non-treated (0 h) and 3, 6 and 12 h after injection of LPS were isolated; and western blotting of claudin-1, -3, -4, and -7 was performed. Beta-actin (mixture of equal parts of the detergent-soluble and -insoluble fractions) was used as the normalization control. (B, D, E) The bands of the insoluble fractions were analyzed by densitometry. (C) The ratio of the upper band to the lower band of insoluble fractions of claudin-3 was calculated using the formula S/P. Data are represented as mean (SD) (n  = 4). *, p<0.05; **, p<0.005 vs. 0 h.

Claudin-1 was not detected in the detergent-insoluble fractions although its presence in the detergent-soluble fractions rapidly increased 12 h after LPS injection (Fig. 7A). The total detergent-insoluble fractions of claudin-3, including both the upper and lower bands, significantly increased 3 h after LPS injection and then significantly decreased 6 and 12 h after LPS injection (Fig. 7B). The ratio of the upper band to the lower band of claudin-3 in the insoluble fractions was also examined. This ratio in the detergent-insoluble fractions of claudin-3 drastically decreased 3 h after LPS injection and was less than 0.1 after 12 h (Fig. 7C). The detergent-insoluble fraction of claudin-4 increased 6 h after LPS injection, and a significant increase was identified after 12 h (Fig. 7D). The detergent-insoluble fraction of claudin-7 was barely detected before LPS injection (Fig. 7E). Three hours after LPS injection, the detergent-insoluble fractions of claudin-7 drastically increased and then somewhat decreased 6 and 12 h after LPS injection. LPS injection induced distinct changes in the detergent-insolubility of claudin-3, -4, and -7. On the other hands.

### TJs are Maintained Around the Alveolar Epithelial Cells during their Shedding Process

**Figure 8:**
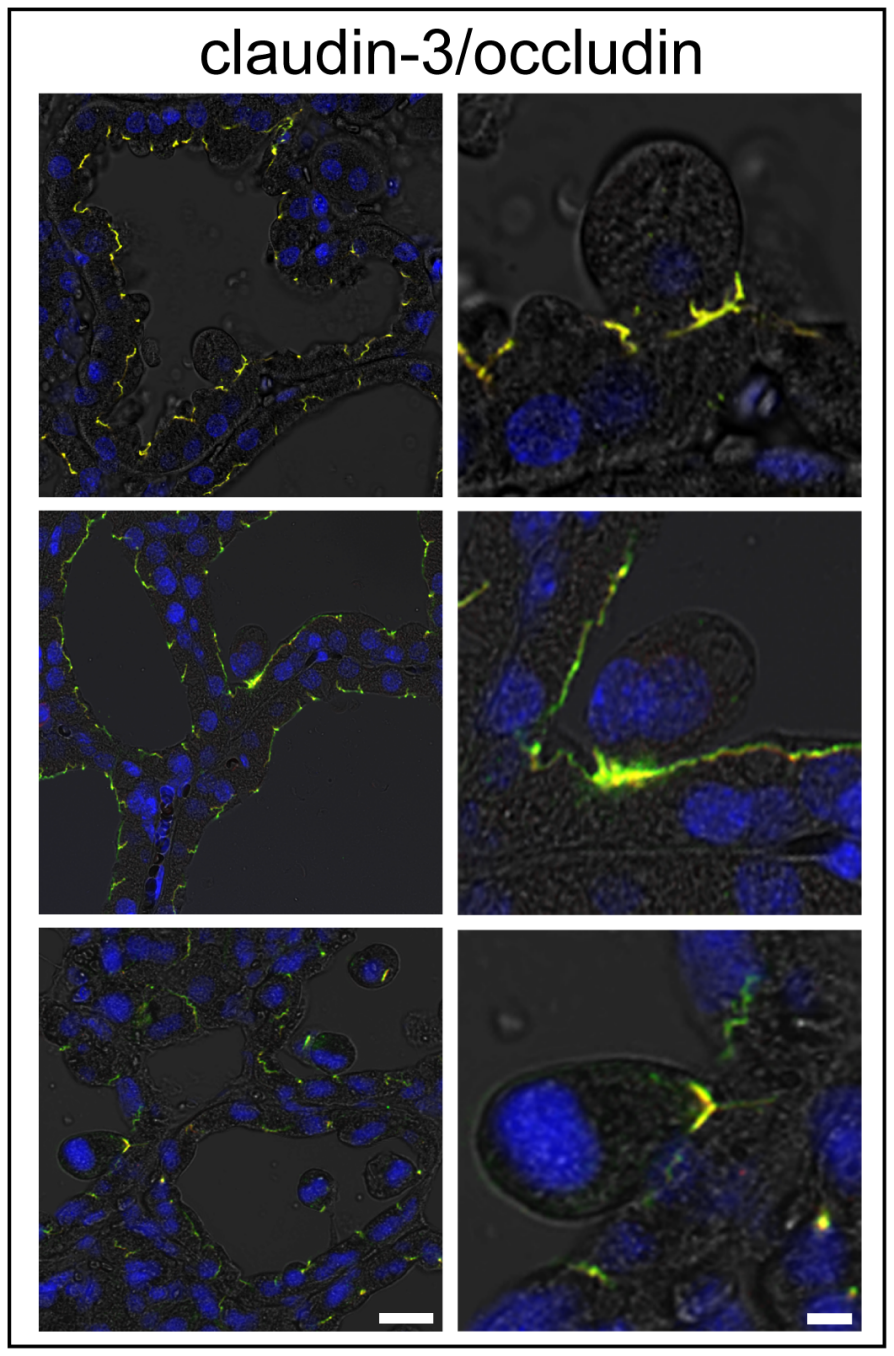
Influences of LPS on shedding of alveolar epithelial cells. Tight junctions (TJs) around shedding cells from the alveolar epithelium by LPS injection were observed by immunostaining for claudin-3 (green) and occludin (red). Continuous TJs were observed between detaching cells and undetached cells during the shedding process in mammary glands 3 (top), 6 (middle), and 12 h (bottom) after LPS injection. Scale bars: 20 µm (left column) and 5 µm (right column).

LPS injection induces the shedding of alveolar epithelial cells into the lumen. To confirm whether TJs become discontinuous at the interfacing regions between the shedding and stable alveolar epithelial cells, the localizations of claudin-3 and occludin were examined as TJ markers because both claudin-3 and occludin participated in the alveolar TJs in mammary glands non-treated and 3, 6, and 12 h after LPS injection (Fig. 4). Claudin-3 and occludin were colocalized at the interface between shedding cells and stable alveolar epithelial cells, indicating that the shedding of alveolar epithelial cells did not induce discontinuous TJs (Fig. 8).

### TLR4 Localizes in the Alveolar Epithelial Cells and LPS Activates the NFκB Pathway

**Figure 9:**
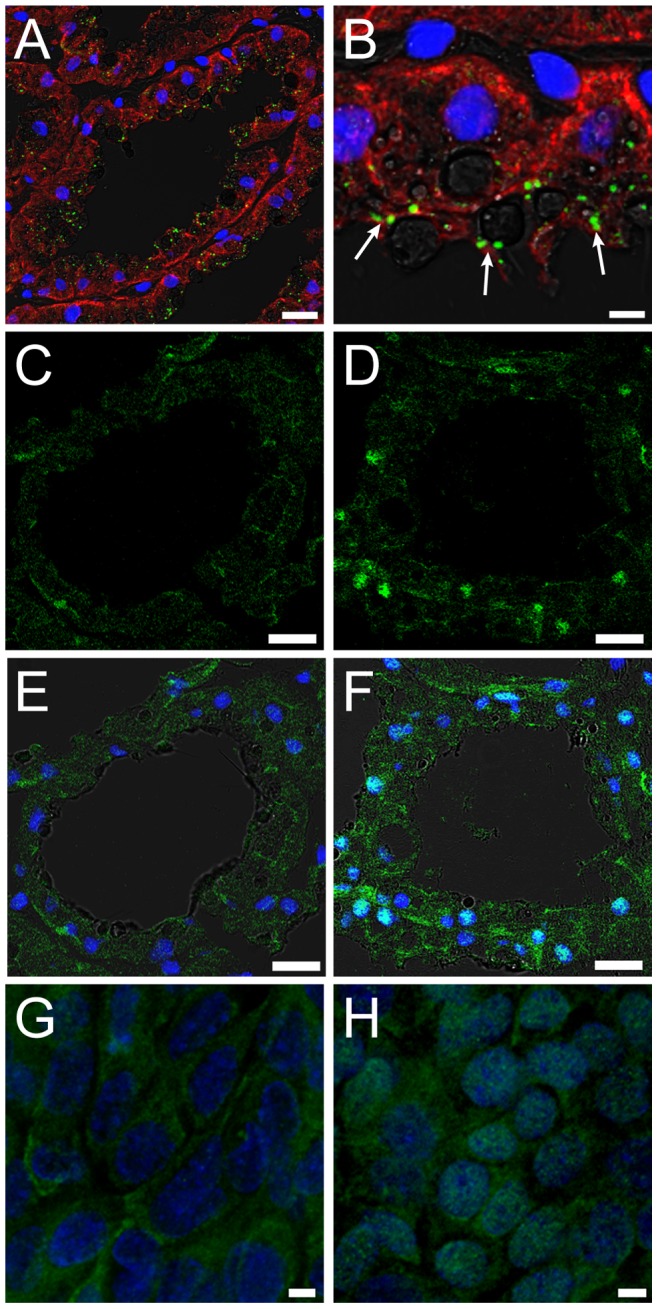
Localization of TLR4 and NFκB in mammary alveolar epithelial cells. (A, B) The mammary glands on day 10 of lactation were immunostained for TLR4 (green) and pan-keratin (red). The localization of TLR4 in the apical membrane was observed (arrow). (C, D) Immunostaining images of NFκB (green) in the mammary gland non-treated and 3 h after LPS injection, respectively. (E, F) Merged images of C and D, with DAPI and bright field, respectively. Cultured mammary epithelial cells without (G) or with LPS treatment for 1 h (H) were immunostained for NFκB (green). Scale bars: 20 µm (A, C–F) and 5 µm (B, G, H).

To confirm whether mammary alveolar epithelial cells can bind to LPS, the localization of TLR4, a representative LPS receptor, was investigated. TLR4 was localized in the alveolar epithelial cells in lactating mammary glands (Fig. 9A). Magnified images showed the localization of TLR4 along the luminal surfaces of the alveolar epithelial cells (Fig. 9B).

NFκB is a downstream molecule of the LPS/TLR4 pathway, and LPS bound to TLR4 stimulates the translocation of NFκB from the cytoplasm to the nucleus [35]. In normal lactating alveolar epithelium, NFκB was localized in the cytoplasm (Fig. 9C, E). Three hours after LPS injection, parts of the alveolar epithelial cells showed the nuclear localization of NFκB (Fig. 9D, F). Such translocation of NFκB was also observed in isolated mammary alveolar cells 1 h after LPS injection *in vitro* (Fig. 9G, H). These results suggest that LPS in the alveolar lumen directly binds to the alveolar epithelial cells and activates the NFκB pathway via TLR4.

## Discussion

Alveolar epithelial cells form highly impermeable TJs that block the leakage of milk and interstitial fluid, but the blood-milk barrier formed by alveolar TJs becomes leaky in mastitis [36]. In this study, the weakness of the blood-milk barrier was recognized as soon as 3 h after LPS injection as the leakage of FITC-albumin into the alveolar lumen. This result coincides with a result obtained by Lehmann that intramammary infusion of LPS disrupts the blood-milk barrier 3 h after injection, as evidenced by an elevation of lactate concentration in blood and IgG concentration in cow’s milk [37]. The shedding of alveolar epithelial cells was also observed 3 h after LPS injection. However, TJs existed at the interface between shedding cells and stable alveolar epithelial cells, indicating that the shedding of alveolar epithelial cells did not disrupt the blood-milk barrier. Therefore, to identify changes in the alveolar TJs induced by LPS injection, we focused on the claudin composition, which is the predominant factor that determines the permeability of TJs [17]. Three hours after LPS injection, claudin-7 participated in TJs as judged by its localization at the apical-most regions and its detergent-insolubility property. Claudin-3 showed a qualitative change as a decrease in the size of the upper band by western blotting. Previously, we reported that the upper band of claudin-3 is detected during lactation but disappears around parturition and after weaning in mice [22]. The phosphorylation/dephosphorylation of claudins influences the permeability of TJs, and seven conserved phosphorylation sites in claudin-3 have been reported [38], [39]. In particular, claudin-3 phosphorylated at threonine 192, which is detected as the lower band compared to nonphosphorylated claudin-3 by western blotting, weakens the barrier function of TJs [40]. These findings suggest that LPS weakens the blood-milk barrier in only 3 h through the participation of claudin-7 in alveolar TJs and qualitative changes in claudin-3, which occur presumably by phosphorylation.

A severe disruption of the blood-milk barrier was recognized 12 h after LPS injection by ectopic localization of β-casein and much leakage of FITC-albumin into the alveolar lumen. In this period, claudin-1 and -4 expressions significantly increased. In particular, claudin-4 was found to participate in alveolar TJs with claudin-3 and -7 12 h after LPS injection from the results of immunostaining and western blotting of the detergent insoluble fractions. Claudin-4 at the apical-most regions appears in the leaky alveolar TJs before parturition and after weaning with qualitative changes in claudin-3 in mice [22], [31], [41]. In addition, induced claudin-4 expression by *E. coli* infection with a weakened TJ barrier has been reported in the bladder epithelium in mice [42]. On the other hand, claudin-1 was localized at the TJ regions with occludin although the participation of claudin-1 in TJs was not recognized by our detergent-insolubility analysis. In normal mouse mammary glands, claudin-1 is not localized in the alveolar epithelium but in the ductal epithelium before parturition and during lactation (unpublished data) [22]. Recently, an increase in claudin-1 expression after discontinued suckling was reported in mice [29]. Claudin-1 is known as an anti-apoptotic factor and tumor suppressor as well as a tumor enhancer/facilitator in breast cancer cells [43], [44]. However, some of shedding cells were claudin-1 positive (data not shown). The role of claudin-1 in this study remains unclear. However, it is suggested that TJs consisting of claudin-1, -3, -4, and -7 are functionally different from the normal lactating alveolar TJs and are leaky, similar to weaning alveolar TJs.

LPS is the specific ligand for TLR4 and binding of LPS to TLR4 stimulates the translocation of NFκB from the cytoplasm to the nucleus [35]. NFκB activation of LPS via TLR4 in the mammary epithelium has also been suggested in *in vivo* and *in vitro* experiments in mice and cows [45], [46], [47], [48]. In this study, localization of TLR4 on the luminal surface of alveolar epithelial cells and activation of NFκB by LPS treatment in cultured alveolar epithelial cells were observed. These reports and our results indicate that LPS binds to TLR4 and induces NFκB activation in the alveolar epithelial cells. The activation of the NFκB pathway increases TJ permeability through localization or expression changes of claudins in rodents and in human epithelial cell lines [49], [50], [51], [52]. In the mammary gland, NFκB activity increases during pregnancy, decreases during lactation, and increases again during involution, whereas the alveolar TJs tighten during lactation in mice and cows [31], [53], [54]. NFκB activation is also observed in *E. coli*–induced mastitis through *in vivo* imaging in mice [46]. Thus, it is suggested that NFκB activation via LPS/TLR4 signaling in alveolar epithelial cells weakens the alveolar TJs with claudin compositional changes in mastitis. Furthermore, the levels of IL-1β, IL-6, and TNF-α increase in mammary glands with *E. coli* infections in mice [55]. LPS rapidly induces the increase in mRNA expression for IL-1β, IL-8, and TNF-α in only 2 h in cultured bovine mammary epithelial cells [56]. These inflammatory cytokines have been reported in inflammation-related disruption of TJs in the intestinal epithelium [57], corneal epithelium [58], epidermis [59], and blood-brain barrier [60]. On the other hand, these cytokines are also secreted from macrophages by LPS treatment, and the involvement of macrophages in *E. coli* invasion has been reported [61], [62]. To further complicate matters, each inflammatory mediator mutually activates the production of another [63], [64]. Such variable factors may cause the complicated changes of claudin composition in direct and indirect ways in mastitis.

In summary, our results show that composition changes of claudins at TJs occurred concomitantly with the disruption of the blood-milk barrier by LPS injection without the collapse of the alveolar epithelial wall by shedding of the cells. Therefore, we suggest that the weakness and disruption of the blood-milk barrier is caused by compositional changes of claudins in alveolar epithelial TJs. Mastitis is an infectious disease of the mammary gland in breast-feeding women, and it is the most costly, common disease and causes a crucial economic loss for the dairy industry [65]. Observations in this study suggest the possibility that the disruption of the blood-milk barrier could be prevented by controlling claudin composition. However, many aspects of the disruption of the blood-milk barrier remain unclear, including the involvement of other claudin subtypes, the regulation of pathways that determine claudin behavior, and roles of inflammatory cytokines that are induced by LPS. Further investigations are required.

## Supporting Information

Figure S1 **Localization of claudin-1, -3, -4, and -7 in the mammary glands treated with PBS injection.** The left column shows the immunostaining images of claudin-3 (green) and nuclear staining with DAPI (blue) in mammary glands 12 h after PBS injection. The middle and right columns show the merged images with occludin (red) and bright field. Scale bars: 20 µm (left and middle columns) and 5 µm (right column).
(TIF)
