# Supplementary figures and images for: Lipopolysaccharide Disrupts the Milk-Blood Barrier by Modulating Claudins in Mammary Alveolar Tight Junctions
Source: PLoS One. 2013 Apr 23;8(4):e62187. doi: 10.1371/journal.pone.0062187 (PMC3633878; doi:10.1371/journal.pone.0062187)

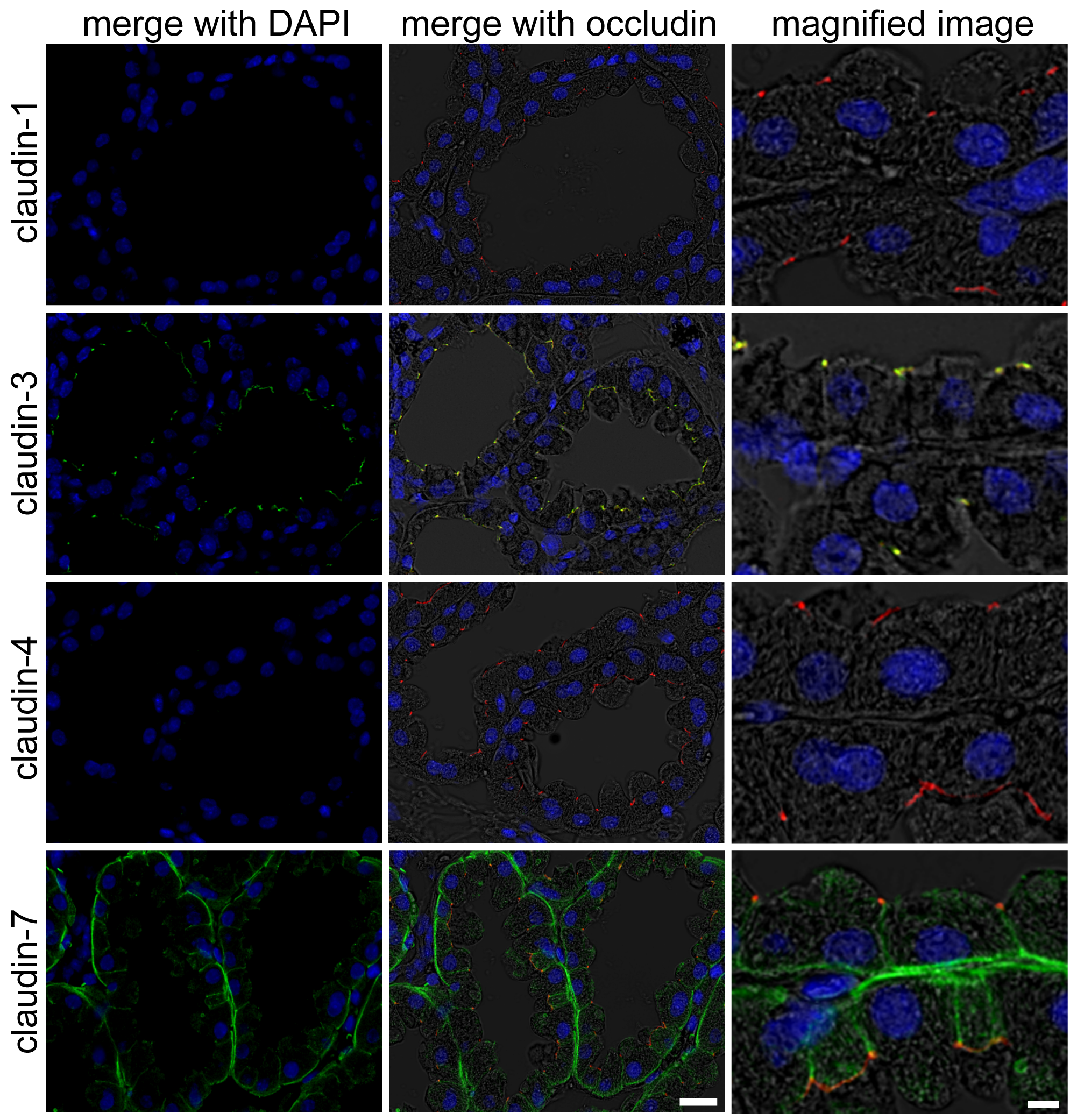

Supplement: Figure S1 — Localization of claudin-1, -3, -4, and -7 in the mammary glands treated with PBS injection. The left column shows the immunostaining images of claudin-3 (green) and nuclear staining with DAPI (blue) in mammary glands 12 h after PBS injection. The middle and right columns show the merged images with occludin (red) and bright field. Scale bars: 20 µm (left and middle columns) and 5 µm (right column). (TIF) [file pone.0062187.s001.tif]
